# The Insidious Cardiac Tumor: A Primary Left Atrium Intimal Cardiac Sarcoma in a Young Patient

**DOI:** 10.1155/2019/7245676

**Published:** 2019-04-04

**Authors:** Chika-Nwosuh Ogechukwu, Nnaoma Christopher, Sossou Christoph, Okundaye Etinosasere, Bustillo Jose

**Affiliations:** Department of Medicine, Newark Beth Israel Medical Center, Robert Wood Johnson Barnabas Health, Newark, NJ, USA

## Abstract

This case presentation discusses a rare cardiac malignancy initially thought to be a benign tumor. A 36-year-old presented with syncope, dyspnea, Computed Tomography Pulmonary angiography study obtained was negative for pulmonary emboli but revealed a left atrial mass. A transesophageal echocardiogram (TEE) confirmed a mass with multiple lobes and a broad base attached to the septum, encroaching into the right atrium, aortic root wall, base of the anterior mitral leaflet flowing to the mitral orifice in diastole also obstructing the right pulmonary vein. Despite a quick diagnosis and plan to begin treatment, the patient rapidly declined owing to the extent and aggressive nature of this cardiac malignancy. This case reports the insidious nature of these tumors as well as how challenging and life threatening they are at the time of their clinical manifestation.

## 1. Introduction

Primary cardiac sarcomas are rare clinical entities and pose challenges in their management, given their aggressive nature and high recurrence rate [1]. Over 75% of primary cardiac tumors are benign, the remaining 25% are malignant [1]. Of the 25% of malignant cardiac tumors, 75% are sarcomas [2]. Up until 2006, fewer than 20 cases had been reported about cardiac sarcomas [1]. Intimal sarcoma also tends to present in the right atrium, albeit rare, they can be seen in the left atrium [3]. The diagnosis relies mainly on image modalities such as echocardiography, computed tomography, coronary angiography, and magnetic resonance imaging. Without tissue sampling, they could easily be mistaken for myxoma or a clot which could delay management [4]. Immediate diagnosis is essential to avoid cardiac complications such as arrhythmias, ischemia due to obstruction of coronary arteries, heart failure, sudden cardiac death, metastasis, or delay in management [5]. Treatment involves resection, chemotherapy, and radiation, with the median survival of six months [6]. Surgical resection remains the most common treatment for most cardiac tumors and involves the complex cardiac reconstruction especially in malignant cases [4].

## 2. Case

A 36-year-old male with no past medical history presented to the hospital with two syncopal episodes, worsening palpitations, and progressive dyspnea on exertion (NYHA class II-III). He reported no prodromal symptoms, immediately regained consciousness after a few seconds without any postictal signs. First syncopal episode occurred 2 weeks earlier while in a passenger seat; he sought no medical attention at the time. The second episode was during a coughing fit the evening of his presentation. Both episodes were said to be witnessed without any seizure activity. Six months prior to the onset of the syncopal episodes, he began experiencing dyspnea with mild exertion which progressed, limiting his functional activities. He reported a 40 lbs weight loss over six months which he had attributed to his intent to lose weight (BMI 38). He also admitted to night sweats but denied chest discomfort, increased abdominal girth, or leg swelling.

**Figure 1:**
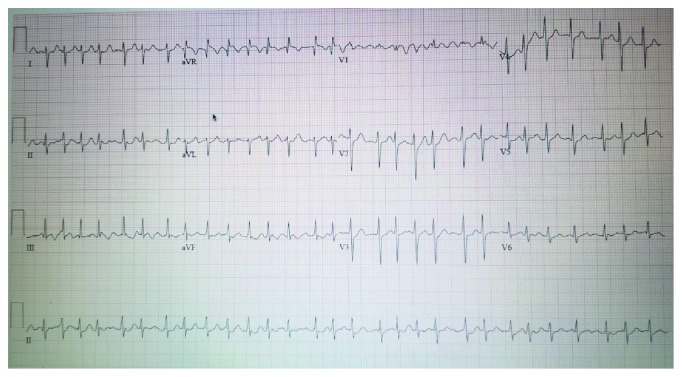
EKG on presentation demonstrating atrial fibrillation with rapid ventricular response.

Physical examination revealed BP 123/78 mmHg, HR 120-150 sbpm which was irregularly irregular, RR 26 breaths per minute, hypoxic at 88% on room air with improvement in Bipap, and laying in Trendelenburg position, negative orthostatic. There was a presence of bibasilar crackles in the lung field, no jugular venous distention or peripheral edema noted, cardiovascular examination was limited due to body habitus and rapid irregular heart rate, and the rest of the exam was unremarkable except for morbid obesity. Electrocardiogram (EKG) showed supraventricular tachycardia and subsequently atrial fibrillation (Figure 1); there was no old EKG to compare. He was started on Cardizem drip at 5 mg/hour and titrated up to maintain a goal heart rate of 80-110 bpm. Chest radiography remarkable for bilateral pleural effusion is worse on the left than the right.

**Figure 2:**
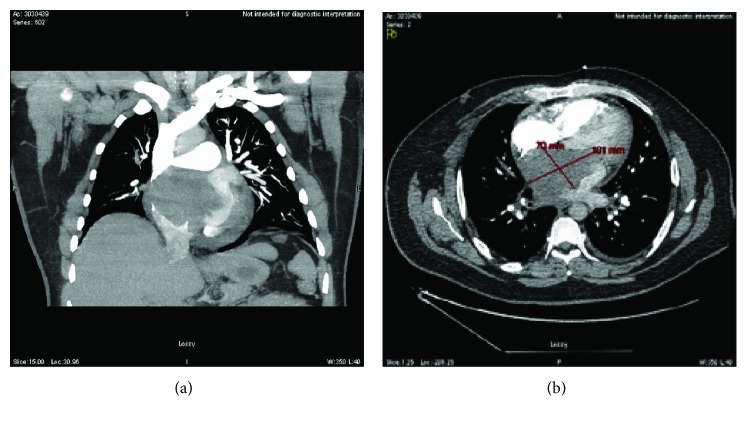
Computed tomography of the chest demonstrating the mass in the left atrium.

**Figure 3:**
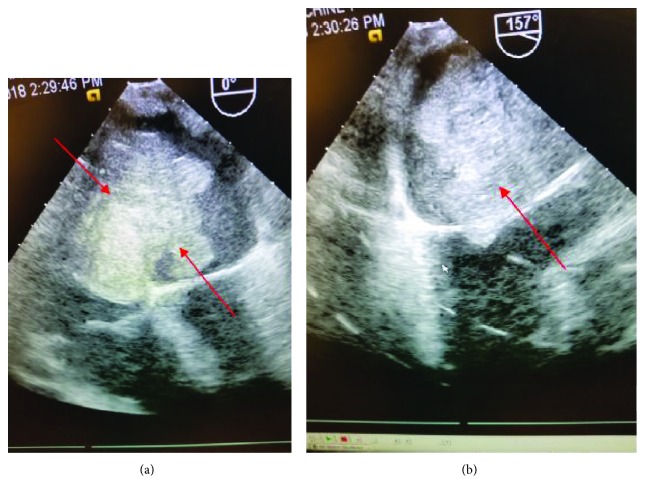
Transesophageal echocardiogram (intraoperative) demonstrating the mass in the left atrium invading the interatrial septum.

A Computed Tomography Pulmonary angiography study was negative for pulmonary emboli but revealed a left atrial mass (Figure 2). A transthoracic echocardiogram (TTE) showed an 8-9 cm atrial mass attached to the interatrial septum (Video 1). Given significant outflow obstruction, the patient was taken to the operating room (OR) for possible resection. Transesophageal echocardiogram (Figure 3) in the OR demonstrated similar mass with multiple lobes and a broad base attached to the septum, encroaching into the right atrium, aortic root wall, base of the anterior mitral leaflet flowing to the mitral orifice in diastole also obstructing the right pulmonary vein seen in. A biopsy and partial resection were performed with no much change in size; the patient was not a candidate for complete resection due to the extensive cardiac involvement.

**Figure 4:**
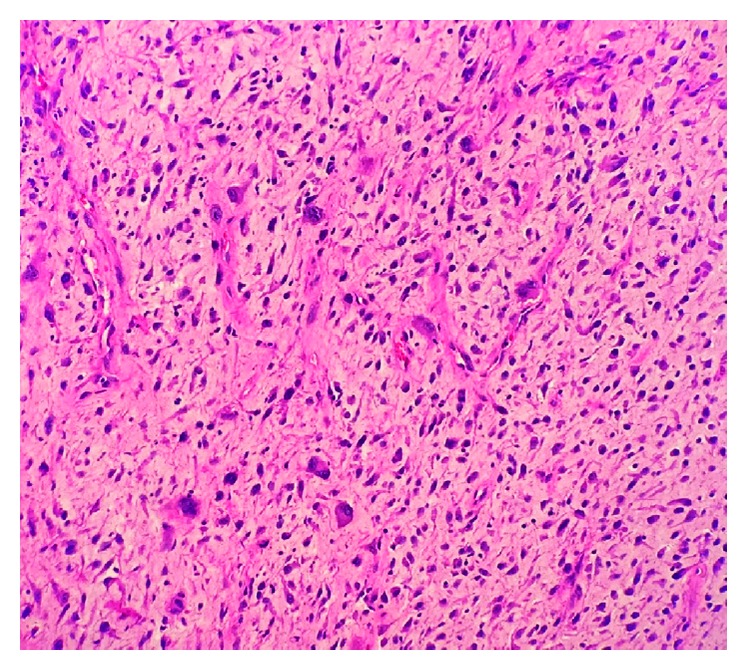
This histopathology illustrates multiple pleomorphic cells (tumor cells) from the partial resection of the left intimal sarcoma.

Pathology (Figure 4) revealed intimal sarcoma evidenced by markers positive for MDM2, CDK4 gene, and KIT and PDGFRA amplification. The patient was rate and rhythm controlled with Cardizem and digoxin. An oncologist was consulted with a plan of treatment with doxorubicin and olaratumab. However, the patient against medical advice wanted to delay treatment for few days. He unfortunately expired from pulseless electrical activity arrest during the same hospital admission after 3 weeks of hospitalization.

## 3. Discussion

Cardiac sarcomas are quite rare, with the male predisposition being 2.5 : 1 [3], and usually occurs in the 3^rd^ and 4^th^ decade of life [3]. It typically occurs in the right atrium and rarely in the left [1]. They often infiltrate into the septum and other structures of the heart resulting in significant outflow obstruction, thus, are very aggressive [3].

In majority of cases, cardiac tumors can be silent; however, when symptoms occur, they are determined based mainly on the size and anatomical location of the tumor [4]. They classically present with signs of congestive heart failure depending on the obstructing atrium. Right heart outflow obstruction can present with jugular venous distention, lower extremities swelling, and ascites mimicking symptoms of right sided heart failure. Involvement of the left side of the heart typically presents with dyspnea due to pulmonary congestion, syncope, and cardiogenic shock from lack of forward flow. Most times, these sarcomas can be confused with benign conditions such as myxoma which are more common tumors of the left atrium [6].

Image studies such as TTE and transesophageal echocardiography, computed tomography, coronary angiography, and magnetic resonance imaging are used for diagnosis and guiding surgical intervention [4]; however, specific diagnosis is made by tissue biopsy and histopathology.

Surgical resection is possible in about 33% of these cases; however, they have a tendency to recur, while others are deemed nonresectable [3]. The standard of treatment involves resection with chemotherapy and radiation. A study by Bakaeen et al. showed a median survival of 24 months in 27 patients after surgical resection and also better outcome with multimodality treatment [6]. In recent studies, neoadjuvant chemotherapy followed by radical resection has been deemed safe and effective in primary sarcoma on the right heart [7]. This mode of treatment enhances resection and can be translated to improvement in survival [7]. Our patient rapidly deteriorated given a significant poor prognosis due to the extent of the mass coupled with his delayed presentation in seeking medical attention. The delay in medical attention, initiation of chemotherapy, the aggressive nature, and the nonresectable mass all compounded to his rapid decline. Early surgical resection with chemotherapy and or radiotherapy may help improve survival in these patients.

## 4. Conclusion

Given the rarity of these cardiac malignancy, very little studies have been conducted regarding the management of these rare tumors especially in nonsurgical candidates; hence, this calls for a meta-analysis on the outcome of surgical candidates and nonsurgical candidates who are treated with chemotherapy and or radiotherapy. This case also calls to question the differences between the left and right atria sarcoma in terms of their presentation, management, and prognosis.

## Supplementary Materials

Supplementary Materials Video 1: transthoracic echocardiogram video showing the mass attached to the mitral valve obstructing inflow into the left ventricle.
